# Infection screening in newly arrived Afghan sanctuary seekers – a family-centred approach

**DOI:** 10.1186/s12913-026-14864-w

**Published:** 2026-07-02

**Authors:** Oluwasomidotun O. Idowu, Paola Cinardo, Humayra Chowdury, Iona Minty, Bernadette Carroll, Nicky Longley, Sarah Eisen

**Affiliations:** 1 Children and Young People’s Division, University College London Hospitals NHS Foundation Trust, London, UK; 2 Business Intelligence Analysis, University College London Hospitals, London, UK; 3 Infectious Diseases, University College London Hospitals, London, UK; 4 Infectious Diseases and Travel Medicine, Hospital for Tropical Diseases, London, United Kingdom; 5 Faculty of Infectious and Tropical Diseases, London School of Hygiene & Tropical Medicine, London, United Kingdom; 6 Great Ormond Street Institute of Child Health, University College London, London, UK; 7 Children and Young People’s Division and Hospital for Tropical Diseases, University College London Hospitals NHS Foundation Trust, London, UK

**Keywords:** Migrant health, Infection screening, Family-centred care

## Abstract

**Background:**

The fall of Kabul to Taliban rule in 2021 placed Afghan citizens working as Locally Employed Staff at risk of threat to life. Approximately 15,000 Afghan citizens were evacuated to the UK under the Afghan Relocation and Assistance Policy (ARAP). Given the urgency, many did not undergo pre-entry screening for infections. The Respond Family Infection Clinic was developed to support the needs of this population.

**Method:**

All sanctuary-seekers who arrived under the ARAP and attended the Respond Family Infection Clinic for screening between November 2021 to March 2023 were included. Family units were defined as having at least one family member who arrived via ARAP. Individual clinical data was extracted from electronic healthcare records and manually grouped into family units. Data was analysed using Microsoft Excel (16.47).

**Results:**

587 Afghan sanctuary-seekers within 94 family units were screened in the study period. 216 infections were identified across the cohort. 17 family units (18.1%) had at least one infection detected within the unit and 55 family units (58.5%) had multiple infections detected (multiple of the same infection and/or different infections). *Giardia lamblia* was the commonest infection identified (57.9% of individuals) in our study population. There was significant prevalence of tuberculosis infection amongst adult individuals (19.1%) and family units (35.1%), as well as Hepatitis B infection (2.2% adults and 4.3% family units). All individuals with positive results were offered treatment.

**Conclusion:**

We describe a family-centred infection screening and treatment service for sanctuary-seekers in North Central London. Our results show significant prevalence of infection in both individuals and family units with potential consequences for personal and public health. Given the prevalence of infections affecting multiple members of family units, provision of care to families in a single appointment with simultaneous management of household contacts reduces the number of appointments required and mitigates the risk of reinfection.

## Background

Following the withdrawal of the North Atlantic Treaty Organisation (NATO) forces from Afghanistan in May 2021, the Taliban mounted a military offense against the Islamic State of Afghanistan. In August 2021, Kabul fell, and the Islamic Emirates of Afghanistan was reinstated under Taliban rule [1].

Even prior to the fall of Kabul, there was recognition that Locally Employed Staff (LES), individuals working with or alongside the UK government or British military in Afghanistan, could be at risk of threat to life due to their work. This risk was further increased after the fall of Kabul [2]. A rapid response was implemented to ensure the safety of Afghan citizens who worked as LES. The Afghan Relocations and Assistance Policy (ARAP) was introduced on 1^st^ April 2021, enabling eligible LES to permanently relocate to the UK, with their families, if they met criteria outlined by the Ministry of Defence and Home Office [2]. Consequently, approximately 15,000 Afghans were evacuated by air to the UK under Operation Pitting where they were placed in government funded temporary accommodation [3, 4].

### Screening for communicable diseases in migrant populations

Due to multiple factors, those seeking sanctuary in the UK from overseas are often at significant risk of infections which may have potential consequences for both individual and public health [5]. Several guidelines exist advising healthcare professionals on the screening of migrants arriving to the UK. The purpose of screening is to detect both symptomatic and asymptomatic infections which are of personal and public health significance [5, 6]. In recognition of the large numbers of Afghan migrants that have arrived in the UK, and the likely additional risk of infection in this group due to the impact of ongoing political instability and conflict on local healthcare services, additional guidance by the UK Health Security Agency (UKHSA) and UK Office for Health Improvement and Disparities (UK OHID) was created [7–9]. Table 1 summarises the recommended screening protocol.

**Table 1:** Recommended infections for screening in newly arrived Afghan refugees [7–18]

Infection	Rationale	Recommended screening			
Tuberculosis (TB)	High incidence in Afghanistan (40 to 499 cases per 100,000) Risk to both individual and public health	**TB disease**	History ^a^	Physical examination	Investigation
		Adults and children over 11	Required	Required if clinically indicated from history	Chest x-ray
		Pregnant women	Required	Required if clinically indicated from history	Chest x-ray in 2^nd^ or 3^rd^ trimester if clinically indicated and with appropriate shielding
		Children under 11	Required	Required if clinically indicated from history	Not required
		**TB infection**			
		All ages and pregnant women	NA	NA	Interferon gamma release assay (IGRA)
Hepatitis B virus (HBV)	Intermediate incidence in Afghanistan (>2% prevalence of chronic Hep B)	Serological testing to be offered to all pregnant women and considered for everyone else *Antigens = HBsAg, HBeAg,* *Antibodies = anti-HBc, anti HBc IgM, anti-HBs, anti-HBe*			
Hepatitis C virus (HCV)	Higher incidence in Afghanistan compared to the United Kingdom	Serological testing (anti-HCV antibody) to be offered if risk factors present^b^ If positive, testing for Hep C virus RNA to confirm the presence or absence of acute infection/re-infection.			
HIV	As part of general health screening and biosecurity.	As HIV rate < 1%, HIV antibody and antigen testing recommended in high-risk groups ^c^ or people registered in a high prevalence area.			
Helminths	Risk of helminth infection amongst Afghanistan migrants	Measure eosinophils (>0.4 × 10^9^ per litres considered positive and possibly suggestive of helminth infection) Consider strongyloides serology Stool microscopy to investigate soil transmitted helminthiasis			
Sexually transmitted infections (STI)	As part of general health screening and biosecurity.	All sexually active people under 25 years old to be screened for chlamydia regardless of symptoms using Nucleic Acid Amplification Test (NAAT)			

^a^History to include: previous TB, cough > 3 weeks, night sweats, unexplained weight loss and fever, haemoptysis, known contact with a case of active pulmonary TB in the last two years within a shared enclosed space

^b^Examples include: previous and current intravenous drug users, living in hostels or homeless, looked after children, babies and children born to mothers with Hepatitis C, HIV positive men who have sex with men, close contact, family member or sexual partner has confirmed Hepatitis C

^c^Sexual contact with an individual from a high HIV prevalence country, clinical concerns of HIV infection, diagnosed with or request testing for STI, sexual partner known to be HIV positive, men who have sex with men or sexual contact with men who have sex with men, history of intravenous drug use, infants and children with HIV positive parents and/or siblings

### Establishment of the family infection clinic

Given the urgency of evacuation, the vast majority of Afghan sanctuary seekers arriving in the UK did not undergo pre-entry screening [7]. The unexpected and significant number of arrivals has posed challenges for NHS services trying to adapt and deliver appropriate services to meet the needs of this population [19].

The Respond Family Infection Clinic was established as a service to screen for and treat communicable infectious diseases in sanctuary-seeking families, many of whom were accommodated in the immediate vicinity of University College London Hospital.

All sanctuary-seekers are eligible to be seen in the clinic. Referrals are accepted either from primary care or opportunistic encounters following presentation to the Emergency Department and/or other services. Screening takes place in a secondary care setting and is provided by paediatric and adult infectious disease clinical nurse specialists (CNS). Families are given the option to attend either as a family or individually. Translation services are offered via telephone with trained interpreters. A basic history and examination are performed to cover allergies, medications, and vaccination status. Sanctuary-seekers are offered screening for communicable diseases. Treatment, if required, is provided at a subsequent appointment, as a family unit if appropriate, or within local services (in the case of tuberculosis infection or disease). All results and treatment are communicated in writing to families and their General Practitioner (GP) to ensure continuity of care.

The panel of infections screened for in the clinic (Table 2) has been adapted from guidance outlined by UKHSA, UK OHID and Royal College of Paediatrics and Child Health [7, 8, 9, 20].

**Table 2:** Infection screening investigations performed at the Respond family clinic at UCLH

Infections screened		Test	Eligibility
Tuberculosis infection		Interferon Gamma Release Assay (IGRA) – QuantiFERON Gold^TM^	Offered to all
Blood Borne Viruses	Hepatitis B virus	HBsAg, anti-HBc, anti-HBs	Offered to all
	Hepatitis C virus	anti-HCV Antibody	
	HIV	HIV antibody and antigen	
Parasites	Strongyloides	Stool microscopy and strongyloides serology	Offered to all
	Helminths	Stool ova, cysts and parasites microscopy and multiplex PCR	
Sexually Transmitted infections	Gonococcal	Urine gonococcal and chlamydia nucleic amplification antigen test (NAAT)	Post pubertal or identified safeguarding risk
	Chlamydia		
	Syphilis	Syphilis serology	Offered to all (given risk of congenital infection in those sexually active)

Chest X-rays are only offered to those who are IGRA positive and/or symptomatic for TB disease (i.e. active TB) or have a history of known exposure to TB disease.

## Rationale for service evaluation

### Aim

We aimed to conduct a service evaluation to understand the prevalence of infection detected amongst Afghan sanctuary-seekers, who arrived via ARAP, using a family-centred infection screening approach.

## Methods

### Study population

Afghan sanctuary-seekers of all ages, who arrived in the UK via the ARAP scheme and attended the Respond Family Infection Clinic between November 2021 and March 2023 were eligible. Family units were defined as participants within the same family group, seen together in one appointment, where one or more family member arrived via the ARAP scheme. Children were defined as any person less than 18 years of age.

### Data collection and analysis

Routinely collected clinical data from Respond Family Infection Clinic service users was reviewed using the electronic healthcare records (EPIC) data collection tool. SQL programming language was used to code and extract the required variables into an encrypted Excel csv file onto a secure network. Family units were not linked via the electronic patient records data collection tool and were therefore grouped manually by the study team. All data were anonymised prior to analysis, with a unique identifying number (UIN) created for all participants. The key linking UINs and patient identifiers were stored on a secure NHS server, within an encrypted file. Data was cleaned using Microsoft Excel (version 16.47) alongside descriptive analysis of quantitative data.

### Ethics approval and consent to participate

This work was deemed a service evaluation, rather than research, by local audit and clinical governance processes according to Trust policy. Ethics approval was therefore not required and the Helsinki Declaration does not apply. As only routinely collected clinical data was analysed as part of this evaluation, individual consent was not required.

(https://www.ucl.ac.uk/research-ethics/do-i-need-ethical-approval#When%20don%92t%20I%20need%20to%20apply)

## Results

### Study population characteristics

587 individuals, comprised of 94 families, attended the clinic between November 2021 and March 2023. The majority of sanctuary-seekers referred to the family clinic were identified following opportunistic encounters with UCLH health services, predominately the Emergency Department. All adults from the 94 families accepted to be seen and screened for infections, in a single family-centred appointment. None preferred an individual appointment. The demographics are summarised in Table 3.

**Table 3:** Baseline characteristics of service users

Characteristics	n
Male	292/587 (49.7%)
Female	295/587 (50.3%)
Adults (>18y)	208/587 (35.4%) *median age 35y (IQR*^*a*^*27-42y)*
Children (<18y)	393/587 (67%) *median age 8y (IQRa 4.5-12y)*
Family units	94 units *median family size 6 people (IQR*^*1*^*4–8)*
Duration of time in the UK^2^	338 days (IQR^b^ 290 - 405)

^a^Interquartile range

^b^Duration of time in the UK between arrival and appointment based on 393/587 participants for whom data was available

### Prevalence of infections

Of those tested between November 2021 and March 2023, 216 infections were identified across the cohort. 37% of these infections were identified in adults and 63% in children. When analysed as family groups, 17/94 (18.1%) of family units had only one infection detected within the unit and 55/94 (58.5%) family units had multiple infections detected within the unit (multiple of the same infection and/or different infections). *Giardia lamblia* was the most commonly identified infection, affecting 21.3% of the cohort (6.8% of adults, 32.9% children and 58.9% of family units). 35.1% of family units had at least one family member screen IGRA positive however, no individuals meet the criteria for TB disease. 4.3% of families had at least one member with active HBV infection. Aside from the known case of HIV, no new cases were identified. Full infection prevalence data is shown in Table 4.

**Table 4:** Infections identified among adult and child participants, and family units

Disease		Adults		Children		Family units with ≥1 member with a positive result	
		Tested n	Positive n (%)	Tested n	Positive n (%)	Tested n	Positive n (%)
**IGRA positive**		204	39 (19.1)	386	2 (0.5)	94	33 (35.1)
**Hepatitis B virus**		181	4 (2.2)	384	0 (0)	94	4 (4.3)
**Hepatitis C virus**		205	0 (0)	385	0 (0)	94	0 (0)
**HIV**		206	1 (0.5)	383	0 (0)	94	1 (1.1)
**Chlamydia**		199	3 (1.5)	100	0 (0)	91	2 (2.2)
**Gonorrhoea**		199	0 (0)	100	0 (0)	91	0 (0)
**Syphilis**		206	4 (1.9)	384	0 (0)	94	4 (4.3)
**Parasites**	**Giardia lamblia**	190	13 (6.8)	340	112 (32.9)	90	53 (58.9)
	**Ascaris spp.**	190	1 (0.5)	340	0 (0)	90	1 (1.1)
	**Hymenolepis nana**	190	3 (1.6)	340	15 (4.4)	90	16 (17.8)
	**Strongyloides**	207	2 (1)	385	1 (0.3)	94	6 (6.4)
	**Cryptosporidium**	190	5 (2.6)	340	0 (0)	90	5 (5.6)

All individuals diagnosed with an infection were offered treatment in the Family Infection Clinic, or, in the case of TB infection, referred to local TB services.

## Discussion

We present an evaluation of a service set up to screen and treat asymptomatic Afghan sanctuary-seekers who underwent emergency evacuation and were resettled within the vicinity of a North London hospital. Although pre-entry health assessments are recommended for all UK-bound people seeking asylum and refugees, these are often not feasible during an emergency evacuation, as was the case for this cohort [21]. Screening on arrival, therefore, plays a key role in identifying health needs including communicable asymptomatic diseases, with the potential for significant health implications. This screening and treatment is important in preventing development of symptomatic disease and onward transmission of infections, with consequent personal and public health benefit.

Family centred infection screening was accepted by all participants to whom it was offered. A high prevalence of infections were identified at screening. Of the 94 family units screened, 72 family units had one or more infections detected (76.6%). The most commonly identified infection was *Giardia lamblia*, with TB infection, HBV, and other intestinal parasites also identified. No new cases of HIV were detected. All individuals were offered treatment within the service or referred to appropriate services.

Amongst Afghan refugees and migrants, the most prevalent reported infections include TB and parasitic infections (intestinal and bloodborne) [22]. However, most data concerning Afghan refugees and migrants involve a diverse group; consisting of people seeking sanctuary from different regions, traversing different routes and with different experiences, for example being placed in detention centres, all of which have an impact on acquisition of infections [22, 23]. Our cohort is unique to a particular group of people who were airlifted to the UK via Operation Pitting under the ARAP scheme. However, a similar group are Special Immigrant Visa Holders (SIVH) in the USA. Afghan SIVHs refer to individuals who were employed by, or on behalf of, the US government or the US military similar to LES. Afghan SIVHs can either be assisted with travel arrangements or choose to travel independently. A recent cross-sectional study looking at this population showed similar rates of infection amongst SIVHs Afghan adults and children to that shown in our work, with intestinal parasites comprising the majority of infections detected and similar prevalence of TB disease and bloodborne infections detected during screening [24].

The higher prevalence of *Giardia lamblia* found in children compared to adults in this cohort is not surprising, given the likelihood of higher faeco-oral spread in the very young. However, the degree of prevalence in this cohort is striking. A study reporting the prevalence of *Giardia lamblia* amongst asymptomatic unaccompanied children seeking asylum or refugees (CYPSAR-U) screened in London between 2016 - 2022, reported a prevalence of 18% compared to 32.9% of children in our evaluation [25]. Considering that the majority of children in our study were directly airlifted to the UK, posing a lower risk for infection, compared to the often longer journeys of CYPSAR-U, who often traverse multiple countries over a prolonged period to reach their destination, the constrasting rates are perhaps surprising. We postulate that this high prevalence may be reflective of poor water sanitation during and following the fall of Kabul, particularly in the airport vicinity while awaiting evacuation. This hypothesis may be further supported given that another study investigating the prevalence of *Giardia lamblia* in the Afghan population, between 2008 and 2012, reported it as 9%, significantly lower than our findings [26]. In children, chronic *Giardia lamblia* has been shown to affect growth and development, even in those who are asymptomatic [27–29]. Given the high transmissibility of *Giardia lamblia* and risk of re-infection between family members and further onward public transmission, infection poses both personal and biosecurity implications that can be managed with prompt identification and treatment to break the chain of infection.

Although HBV infection was not identified within our paediatric cohort, the significant prevalence amongst family units suggests that unvaccinated children are nonetheless at risk of inter-family infection. Horizontal transmission accounts for most cases of chronic HBV infection in intermediate prevalence areas, especially in early childhood [30]. In addition, considering that a reported history of acute hepatitis occurs only in a small percentage of those with chronic HBV infection, screening is important in detecting asymptomatic cases [31]. The consequences of chronic HBV infection vary from an inactive carrier state to potential risk of liver cirrhosis, hepatocellular carcinoma and extrahepatic complications such as polyarteritis nodosa, membranous nephropathy and aplastic anaemia [31–35]. HBV infection therefore poses risk of serious morbidity and mortality at both an individual and public health level. Detection via screening allows for treatment of active disease and mitigation of the risk of horizontal and vertical transmission via the use of education, anti-virals, immunoglobulins and vaccination where appropriate [36]. Despite the low prevalence of blood borne viruses and other sexually transmitted infections identified in our cohort, the importance of screening for these infections for both personal (including future pregnancies) and public health is likely to outweigh the cost implications of routine testing. Incorporating such tests into a panel, rather than a targeted risk-based approach, has been shown to have several advantages such as avoidance of recall bias, reduction in the number of appointments and promoting a more trauma-informed model of care [25]. In addition, evidence shows that where screening is conducted as part of a general health check-up, stigma may be reduced and uptake often increased [37].

The prevalence of IGRA positive individuals within this cohort is significant (19.1% of adults, 0.5% of children, and 35.1% of family units having at least one member screen IGRA positive). Although the number of children with positive IGRA results is relatively low; it’s important to recognise that there is a high risk of progression from TB infection to TB disease in the paediatric population [38]. In addition, the significant number of family units with at least one member having a positive IGRA result is suggestive of significant risk of household exposure. In healthy adults; the annual risk of progression from TB infection to TB disease is 0.1%. This risk is influenced by other diseases and lifestyle factors such as immunosuppression, smoking, diabetes mellitus [39, 40]. The physical impacts of TB vary depending on a myriad of factors including site of infection. Complications from the disease can therefore vary and include focal neurological deficit from tuberculoma and impaired pulmonary function from pulmonary TB [41]. However, the impact of TB isn’t just physical. The presence of TB disease and a delayed diagnosis have been associated with negative economic consequences, such as income loss and psychological consequences such as depression and anxiety [42–44]. TB has also been recognised by the UKHSA to be one of the diseases associated with outbreaks in asylum seeking accommodation in the UK [45]. With the potential for progression to active disease and risk of dissemination, early detection by screening allows for appropriate identification of these individuals and prompt management, especially amongst a group of already vulnerable individuals [46, 47].

The Respond Family Clinic provides a “one-stop shop” approach for screening and treatment of infections within families. It improves efficiency by reducing the number of appointments required by seeing multiple affected family members in a single appointment within a single service. The service was able to screen a total of 94 families in 94 appointments over 16 months. In a standard model of care, this would have required 587 individual healthcare appointments, with significant implications for resources, time and financial cost to service users thus potentially impacting attendance. This model of care is likely to reduce school absences and travel costs, improve retention in care, and allows for health education and intervention to be provided to the whole family contemporaneously. Furthermore, treatment of all infected family members simultaneously reduces risk of reinfection of treated members within the family unit and may allow for more efficient disease control.

Although not within the scope of this paper, work from our group has previously demonstrated acceptability of the family clinic model. This work showed that the model was perceived as effective in addressing health needs, and that service users were comfortable being seen together in a family appointment. Although individuals were offered the opportunity to be seen alone, this was not taken up within this cohort. All families reported they had enough time to discuss their needs and appreciated the support and unity they felt from having their family with them.^1^

Similar models of family-centred care have been found to be both acceptable and effective in the screening and management of HIV both in the UK and abroad [48–50]. At a paediatric tertiary care hospital in London, the utilisation of a family model allowed for the detection and ongoing management of HIV amongst undiagnosed family members presenting to hospital with unwell children, later diagnosed with HIV, without the need to access alternative services. The access to both adult and paediatric healthcare within one setting provided the advantages of better co-ordination of care amongst the multi-disciplinary team alongside reduction in expenses, inconvenience and need for child care for families [48]. The utilisation of the family model of care in Nyanza Province, Kenya, provided the benefit of increasing HIV case detection (particularly amongst the asymptomatic), and identifying those at risk of contracting HIV, by identifying and providing access to screening for family members of index cases, with subsequent care offered where required [49]. Qualitative data looking at the acceptability of a family-centred HIV care model at four health facilities in Hhohho Region, Eswatini, found that the model was positively received by families and healthcare workers and felt to improve both the physical and psychosocial aspects of care through strengthening relationships and health information sharing for families. However, there was also recognition that running such a service requires appropriate training of healthcare workers such as training in supporting caregivers to disclose their HIV status to children, managing large groups in one setting, and appointment scheduling challenges amongst families with school-going children or those with extra-curricular activities [50].

Challenges associated with provision of a family service for those seeking asylum include the provision of clinic settings with adequate space for large families, adequate numbers of staff that are trained in trauma informed care, staff that can deliver clinical care to both adult and paediatric populations, and additional administrative staffing with time to provide support for large family groups. There is also a risk that the lack of confidentiality posed by a family appointment may limit disclosure of sensitive or potentially stigmatising information, which may have both health and safeguarding implications. This may be of particular relevance in the context of infections which may be stigmatised, such as HIV. Despite the offer of individual appointments to all those seen in our service, and the universal uptake of the family appointment, we acknowledge that this is a potential risk.

Significant resources are required to ensure accessibility to populations who may encounter barriers to healthcare access, thereby making them vulnerable, as seen amongst people experiencing homelessness [51]. Within our study population, we had no loss to follow-up. This is likely a result of our bespoke administrative processes recognising the vulnerability of those seeking asylum. It includes proactively contacting referred sanctuary seekers, use of telephone interpreters for reminders of upcoming appointment(s), provision of support to attend appointments including financially (e.g. pre-paid travel cards), and a policy of rebooking appointments not attended. This is a resource intensive approach, but appears to have been effective in ensuring engagement with our cohort. Despite the financial implications of providing such a service, it is likely that these may be offset by the longer term benefits of prompt diagnosis of infections, which would cost more in the long term to address if allowed to become symptomatic and, in the case of TB and HBV infection, require a public health response. Although detailed economic evaluation is beyond the scope of this work, given the efficiency of appointment provision achieved by the simultaneous review of all family members, we postulate that this model of care is likely to deliver significant short, medium, and long-term cost savings.

We have demonstrated an effective service for identifying and treating infections in this population. We believe this family model may provide advantages over a conventional model of care. Due to commissioning restrictions, our service was not able to address wider health needs. This is a significant missed opportunity, to address both other physical and mental health issues, and is of particular significance in the context of vaccination. Provision of immunisations in a “one stop shop” approach would provide an efficient approach to address a significant health need in a vulnerable population. This is particularly relevant in the context of HBV infection, which, if diagnosed in one family member, as was the case in several families here, poses significant ongoing transmission risk to other household members. This challenge is reflective of wider systemic challenges across health services [52, 53]. Fragmentation of services between the NHS and social care, and between primary and secondary care, in addition to lack of understanding of care eligibility by both service users and service providers, incompatibility of digital operating services and financial provision across sectors and services creates an environment of disjointed care, long waiting times and access barriers for service users. Consequently, vulnerable individuals are at an increased risk of unmet needs which may led to increased emergency and unscheduled healthcare attendances with potential high loss to follow up and poor health outcomes [52, 54–58]. This is of particular relevance for sanctuary seekers, who often face significant additional challenges in accessing healthcare due to language and cultural barriers; further exacerbating health inequalities [52, 59–62]. Continued advocacy for joined up commissioning, streamlined care and opportunistic provision of immunisations is essential for inclusion health groups [52].

## Limitations

Access to our service was by referral only, from either a primary or secondary healthcare provider. Although inclusivity was promoted by opportunistic identification of eligible individuals attending hospital for other reasons, inevitably the referral criteria, and lack of self-referral mechanism, result in inclusion bias, as only those already engaged in healthcare were identified.

Our results relate to a specific population and clinic setting and are therefore not generalisable. However, they offer valuable insights into the effectiveness of a family infection clinic service in asylum-seeking and refugee populations and into the prevalence of infection in a previously understudied cohort of vulnerable individuals and families.

## Conclusion

The evaluation of a family-centred infection clinic set up to meet the need of newly arrived Afghan sanctuary seekers, via ARAP, at a London hospital has demonstrated a significant prevalence of treatable communicable infections (notably TB infection, HBV, HIV, chlamydia and Giardia), with both personal and public health implications. This is important evidence to underpin development and provision of bespoke services in this vulnerable cohort, both to address infection, and, ultimately, wider health needs.

## Data Availability

The datasets used and analysed during the current study are available from the corresponding author on reasonable request

## Abbreviations

ARAP: Afghan Relocation and Assistance Policy
NATO: North Atlantic Treaty Organisation
UKHSA: UK Health Security Agency
UK OHID: UK Office for Health Improvement and Disparities (UK OHID)
CNS: clinical nurse specialists (CNS)
GP: General practitioner
UIN: unique identifying number (UIN)
CYPSAR-U: children seeking asylum or refugees
TB: Tuberculosis
IGRA: Interferon gamma release assay
HBV: Hepatitis B virus
HCV: Hepatitis C virus
HIV: Human immunodeficiency virus
STI: Sexually transmitted infection

## Footnotes

1 Unpublished data (2023 - Ni Chaoilte A, Sanchez Clemente N, Lilygreen R, Longley N, Eisen S). Work accepted for presentation and currently under review for publication in a peer reviewed journal – reference pending and will be supplied.
